# Effect of dietary and lifestyle intervention in pregnant women: randomized clinical trial

**DOI:** 10.11606/s1518-8787.2025059006259

**Published:** 2025-10-24

**Authors:** Maria Carolina de Lima, Natalia Posses Carreira, Daniela Saes Sartorelli

**Affiliations:** I Universidade de São Paulo Faculdade de Medicina de Ribeirão Preto Departamento de Medicina Social Ribeirão Preto SP Brasil Universidade de São Paulo. Faculdade de Medicina de Ribeirão Preto. Departamento de Medicina Social. Ribeirão Preto, SP, Brasil

**Keywords:** Pregnancy, Randomized Controlled Trial, NOVA Classification, Physical Activity, Diet

## Abstract

**OBJECTIVE:**

To evaluate the intra- and between-group (control and intervention) effect of counseling during pregnancy on pregnant women’s food consumption and physical activity.

**METHODS:**

This randomized, parallel, two-arm clinical trial was carried out in primary health care in a Brazilian municipality from 2018 to 2021. Adult pregnant women with pre-gestational overweight (n = 350) were randomly assigned to the control or intervention groups. The intervention consisted of three counseling sessions based on the NOVA food classification system and encouragement toward physical activity. Then, 24-hour dietary records were obtained, and physical activity was assessed using a structured questionnaire. The Wilcoxon test was used for intra-group differences between assessments and the Mann-Whitney test, for differences between groups.

**RESULTS:**

Women in the control group consumed less minimally and unprocessed foods (%E) (Δ = −4.08; -13.58 to 4.34; p = 0.006) and more ultra-processed foods (%E) (Δ = 3.74; −5.86 to 12.86; p = 0.009), with no difference between groups. The intervention group showed an increase in commuting-related physical activity (min/week) between assessments (Δ = 9.00; −30.00 to 70.00; p = 0.02), with no difference between groups. The other parameters showed no differences.

**CONCLUSIONS:**

The intervention failed to affect outcomes. However, intragroup changes showed that the control consumed less minimally and unprocessed foods and more ultra-processed foods and the intervention group increased their commuting-related physical activities.

## INTRODUCTION

The consumption of ultra-processed foods (UPF) has increased worldwide in recent decades^1^. These cheap industrial dietary energy and nutrient formulations include additives and result from a series of processes. They also include high amount of energy and unhealthy types of fat, refined starches, free sugars and salt, and poor sources of protein, dietary fiber, and micronutrients. Their design target hyperpalatability, large appeal, long shelf life, and the ability of being consumed anywhere at any time^2^.

The consumption of these foods during pregnancy is associated with undesirable effects on the health of the mother-child binomial, including maternal obesity^3^, excessive gestational weight gain (GWG)^4^, gestational diabetes mellitus^5^, arterial hypertension^6^, undesirable biochemical alterations^7^, increased neonatal body fat and risk of cesarean section, large-for-gestational-age newborns, and macrosomia^8^, in addition to the negative impacts on the environment^9^and the implications in the social, cultural, political and economic spheres^10,11^.

Moreover, a sedentary lifestyle during pregnancy is associated with a significant risk of deep vein thrombosis and obesity. The latter is associated with spontaneous abortions, neural tube defects, increased risk of gestational diabetes mellitus, preeclampsia, sleep apnea, macrosomia, premature birth and even stillbirth^12^.

Therefore, conducting lifestyle intervention studies during pregnancy to encourage healthy eating and regular physical activity (PA) are of great importance for maternal and child health. However, few lifestyle intervention studies during pregnancy have been conducted in primary healthcare (PHC)^13,14^ and none based their intervention on the Dietary Guidelines for the Brazilian Population, the current guideline for promoting adequate and healthy eating.

This study aims to evaluate the intra- and between-group (control and intervention) effect of a dietary and lifestyle counseling on food consumption and PA for overweight pregnant women.

## METHODS

### Study Design and Population

This randomized clinical trial was carried out with adult overweight pregnant women attending seven health units in Ribeirão Preto, state of São Paulo, from 2018 to 2021 following CONSORT guidelines. Further details can be found in Sartorelli et al.^15^ Trial registration: Brazilian Clinical Trials Registry (Rebec) RBR-2w9bhc, July 30, 2018.

As recent studies on the effect of interventions on GWG in overweight women used excessive GWG as the primary outcome, such parameter was chosen to determine the sample size of this primary clinical trial^16^. Thus, a minimum significance level of 5% (α = 0.05), a power of 90% (β = 0.1), and a loss to follow-up of 20%^17^ indicated a sample of 300 pregnant women. However, due to the COVID-19 pandemic, the proportion of loss to follow-up in the study totaled 40%, which resulted in a final sample with 350 pregnant women.

Convenience sampling was considered in this study since all pregnant women with available data for the explored outcomes were included in its analyses^18^. A sample of 106 women guaranteed the statistical power to explore the three proposed outcomes, considering a minimum significance level of 5% (α = 0.05), a power of 70% (β = 0.3), and an effect size of 0.5 (as calculated on G Power 3.1.9.2).

Inclusion criteria included women who were aged ≥ 18 years with a gestational age at the time of screening up to the 15th week and six days and a pregestational body mass index (BMI) of 25.0 to 29.9 kg/m^2^, indicating overweight. Pregnant teenagers (those aged 18 and 19 years) (n = 11) were classified according to BMI/age^19^.

Exclusion criteria included multiple gestations, a history of type 2 diabetes, and the use of oral hypoglycemic or weight-loss medication (Figure).

**Figure:**
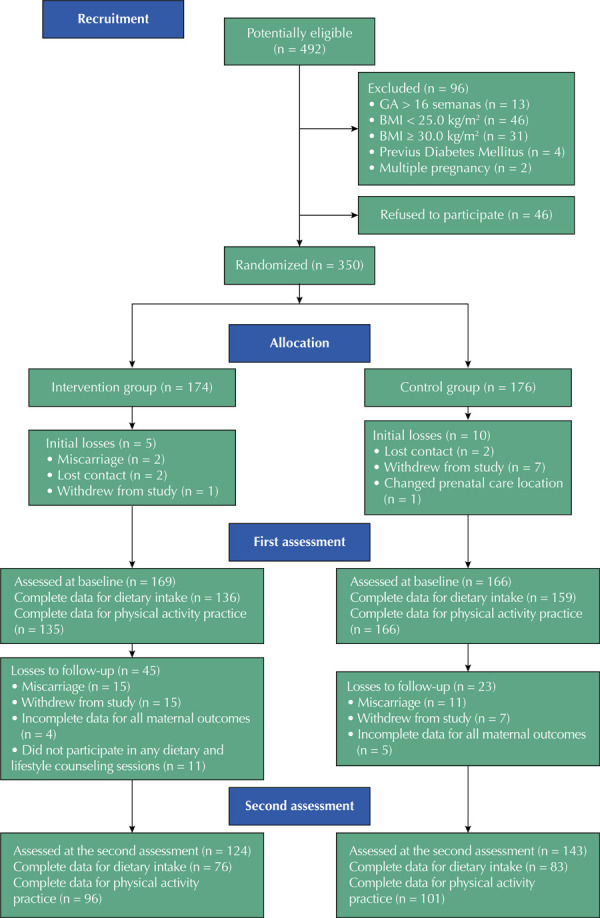
Study flowchart.

### Randomization, Blinding, and Group Allocation

Participants were randomly allocated into the control (CG) and intervention group (IG) by Research Electronic Data Capture (REDCap) software^20^and a spreadsheet of randomly generated numbers. Stratification among groups considered the prenatal health units, equally distributing the number of pregnant women from each health facility to each group. Both participants and researchers were aware of the group allocation due to the nature of the intervention.

### Study Assessments

Participants underwent two assessments during their pregnancy. The first assessment was conducted up to their 15th gestational week and six days (before counseling), whereas the second assessment preferably occurred from the 34th and 36th gestational weeks. Tablets with REDCap software were used for data collection^20^.

Height (meters) was measured during the first assessment using a stadiometer with an integrated mechanical scale available in the health unit. Weight (kilograms) was recorded using a portable digital scale (Tanita, model HS 302) during both assessments. Information regarding age, self-reported skin color, marital status, parity, the education level of participants and the household heads, engagement in paid work, parity, morbidities, frequency of alcohol consumption, use of dietary supplements, and smoking were obtained via a structured questionnaire.

The date of the last menstrual period in the pregnant woman’s prenatal follow-up notebook was used to calculate gestational age, which was later confirmed by ultrasound data up to the 20th GW. The 2019 Brazil Economic Classification Criteria^21^ was used to classify participants’ socioeconomic status. This classification is based on item possession, household head’s education, housing with piped water, and paved streets, categorizing the socioeconomic status from class A (highest level) to class E (lowest level).

### Dietary and Lifestyle Counseling

Standard prenatal care (including prevention, health promotion, and treatment of pregnancy complications) was administered to all participants at their health units. The women in the IG were invited to participate in three individual dietary counseling and PA promotion sessions averaging 30 minutes per session, with a six-week interval, on their prenatal care visits at the health units. The sessions were carried out by a trained dietitian.

Dietary counseling and PA promotion used educational materials consisting of three folders (one for each meeting) containing key messages and images that illustrated the outlined objectives. All counseling topics were addressed in the three sessions by approaches that suited their gestational progress. This educational material was developed and validated before this study^15^.

The women were informed about the goals of the intervention in the first counseling session, which adopted recommendations from the Institute of Medicine^22^, Brazilian Dietary Guidelines^23^, and the American College of Obstetricians and Gynecologists^24^ as theoretical references. Those with obstetric contraindications for PA were advised to follow their physician’s counseling. The guidelines were reinforced in subsequent sessions by different approaches according to the evolution of their pregnancy.

The counseling sessions included the following guidelines:

1. Guidance on adequate total weight gain during pregnancy (from seven to 11.5 kg);
2. Regular weekly physical activity (150 minutes/week);
3. Encouragement of the consumption of unprocessed and minimally processed foods, prioritizing homemade foods to the detriment of the consumption of UPF;
4. Encouragement of water consumption over the consumption of soft drinks and juices.

The other pamphlets can be accessed at Manochio-Pina et al.^25^ (2022).

The average time between the first assessment and the first counseling session equaled 34.7 days and the average time between the last counseling and the second assessment, 44 days. Due to the restrictions caused by the COVID-19 pandemic, interventions planned from March to October 2020 were conducted online via video or voice calls, according to participants’ preferences (IG; n = 13 and CG; n = 11).

After birth, participants received one counseling section to assist them to regain their pregestational weight and encourage breastfeeding.

As the CG received no counseling sessions between the first and second assessments in the study, the researchers’ contact with this group only occurred during assessments.

### Assessment of Dietary Intake

In total, two nonconsecutive 24-hour dietary recalls were obtained by trained nutritionists in the first assessment (averaging 11 days between replicates) and two in the second assessment (averaging seven days between replicates). In the first study assessment, the second 24-hour dietary recall was obtained from 82.9% of participants, and in the second assessment, from 24.2%.

Participants’ reports of all food, preparations, and beverages (with quantities in household measures) they consumed the day before the interview were collected by the seven-stage “multiple pass” methodology to obtain their 24-hour dietary recalls^26^, enabling the classification of foods according to NOVA, which categorizes food items into unprocessed foods (directly obtained from plants or animals without any industrial intervention); minimally processed foods (foods subjected to mechanical processes that add no substance to the original food); processed foods (manufactured by the industry by adding substances to increase their durability and palatability); and UPF (industrial formulations primarily or entirely made from sugar, salt, oils, fats, starches, many substances derived from foods typically absent in kitchens, and cosmetic additives, including those used to imitate the sensory qualities of natural foods or disguise undesirable qualities in the final product)^27^. Culinary preparations, including items from several food groups, were classified based on the most abundant ingredient in the preparation^27^.

The Nutrition Data System for Research was used to estimate dietary nutrients. The food groups were expressed in energy percentages (%E). The Multiple Source Method was used to estimate the usual diet at each assessment point^28^.

### Assessment of Physical Activity

Commuting and leisure PA were evaluated in both study assessments. A questionnaire that captured details about the type, frequency, and duration in these activities as well as screen time throughout the week before the interview was used. It was adapted from previously validated for the adult Brazilian population *Sistema de Vigilância de Fatores de Risco e Proteção para Doenças Crônicas por Inquérito Telefônico* (VIGITEL – Surveillance System for Risk and Protective Factors for Chronic Diseases by Telephone Survey)^29^.

Leisure-time physical activity (LPA) was based on the free time participants spent on recreational activities during the week. Commuting physical activity (CPA) was considered as the minutes/week spent on walking or cycling for transportation. Screen time was assessed by the reported average hours per day watching television or using a computer, tablet, and/or cell phone. The total time spent on LPA and CPA was summed, and the women were classified as meeting the recommended minimum PA/week ≥ 150 minutes or as failing to meet the recommendations of the Physical Activity Guide for the Brazilian Population^30^.

### Statistical Analysis

Data normality was verified by the Shapiro-Wilk test. The modified intention-to-treat analysis was ignored in the original study protocol^15^. However, in-person interviews were suspended due to the pandemic, leading to a greater-than-expected loss of follow-up. As a result, some women in the IG did not participate in any counseling sessions. However, a modified intention-to-treat analysis was adopted excluding participants who did not attend any counseling sessions^31^.

To characterize the study population according to treatment groups, continuous descriptive variables are shown as medians (P25; P75) and categorical variables, as frequencies n (%).

The difference in intra-group medians (Δ) between the assessments for each outcomes was calculated and organized into tables.

The effects of the interventions on the change in food consumption and PA were obtained by the Wilcoxon test to measure intragroup differences between assessments and by the Mann-Whitney test to assess differences between the IG and CG. Data are shown as medians (P25; P75). All analyses were conducted on SPSS, version 21, and the level of significance was set at p < 0.05.

This study hypothesized that dietary counseling and PA promotion may reduce UPF consumption and increase the consumption of minimally processed and unprocessed foods and PA.

## RESULTS

Of the 350 randomized pregnant women, 335 (CG = 166 and IG = 169) completed the baseline assessment. Their median age (P25; P75) equaled 27 (23 to 32) years, whereas their median gestational age at randomization, 11 (9 to 12) weeks and their median pre-gestational BMI, 27.2 (26.1 to 28.3) kg/m^2^. Treatment groups showed no difference in maternal characteristics (Table 1).

**Table 1:** Characteristics of the pregnant women according to treatment group. Ribeirão Preto, SP, Brazil, n = 335.

Characteristics	Intervention (n = 169)	Control (n = 166)	p^a^
	Median (P25; P75)		
Maternal age, years	27 (23 to 31)	27 (22 to 32)	0.45
Gestational age at randomization, weeks	11 (9 to 13)	11 (9 to 12)	0.86
Baseline BMI, kg/m^2^	27.2 (26.2 to 28.3)	26.9 (25.9 to 28.4)	0.38
	n (%)		
Married/cohabiting with partner	132 (78.1)	120 (72.3)	0.22
Self-reported skin color^b^			0.91
White	50 (30.3)	53 (32.3)	
Black	26 (15.8)	24 (14.6)	
Brown/mixed race	89 (53.9)	87 (53.0)	
Education, years			0.61
≤ 8	36 (21.3)	40 (24.1)	
9–11	108 (63.9)	107 (64.5)	
≥ 12	25 (14.8)	19 (11.4)	
Socioeconomic stratum^c^			0.39
A+B	32 (21.2)	31 (21.1)	
C	98 (64.9)	87 (59.2)	
D+E	21 (13.9)	29 (19.7)	
Paid work at randomization	109 (64.5)	90 (54.2)	0.06
Nulliparous^d^	56 (33.1)	66 (39.8)	0.33
Use of dietary supplements	152 (89.9)	148 (89.2)	0.82
Smoking habits			0.09
Never smoked	123 (72.8)	116 (69.9)	
Currently smokes	7 (4.1)	17 (10.2)	
Former smoker	39 (23.1)	33 (19.9)	
Reported alcohol consumption in the last 30 days	31 (18.3)	34 (20.5)	0.62
Gestational diabetes mellitus^e^	17 (12.9)	20 (14.1)	0.77
Pre-gestational hypertension^f^	7 (4.2)	5 (3.0)	0.56

Source: adapted from Sartorelli et al.^15^

^a^ Student’s *t*-test or Mann-Whitney U test for continuous variables or the chi-squared test for categorical variables. ^b^ Self-reported skin color is used as a proxy for ethnicity in Brazil. Only five women reported being of Asian descent and one refused to answer. Analysis excluded them. No women reported being Indigenous.

^c^ According to the Brazilian Economic Classification Criterion, which categorizes socioeconomic conditions from class A (highest level) to class E (lowest level). Data available for 151 women in the intervention group and 147 in the control group, as 37 were unaware of the household head’s education level.

^d^ Data available for 169 women in the intervention group and 165 in the control group.

^e^ Data available for 132 women in the intervention group and 142 in the control group.

^f^ Data available for 167 women in the intervention group and 166 in the control group.

Of the women in the IG, 15 (8.8%) attended one counseling session; 22 (13.0%), two sessions; and 99 (58.6%) three sessions. The median gestational age of women who participated in the first counseling session equaled 15 (13 to 18) GW; in the second session, 22 (19 to 25); and in the third session, 29 (25 to 31). Regarding adherence to regular prenatal care, no difference was found between the groups considering the average number of consultations [CG = 7.42 (± 1.77) and IG = 7.41 (± 1.77)].

Considering the modified intention-to-treat, a reduction in the %E from the usual consumption of minimally processed and unprocessed foods (%E) in the CG (Δ = −4.08; −13.58 to 4.34; p = 0.006) occurred along with a significant increase in UPF (%E) between assessments in the CG (Δ = 3.74; −5.86 to 12.86; p = 0.009). No changes in food consumption occurred in the GI assessments or between groups (Table 2).

**Table 2:** Food groups according to NOVA (%E) consumed by pregnant women, Ribeirão Preto (SP), Brazil, n = 294.

	Intervention^a^	Control^a^	p^b^
	Median (P25; P75)	Median (P25; P75)	
Unprocessed and minimally processed (%E)			
1^st^ assessment	65.02 (57.34 to 72.60)	66.25 (58.32 to 72.69)	0.47
2^nd^ assessment	67.69 (55.06 to 75.29)	63.84 (53.14 to 73.98)	0.24
Δ	0.58 (−6.53 to 15.25)	−4.08 (−13.58 to 4.34)	
p^c^	0.35	0.006	
Processed (%E)			
1^st^ assessment	6.33 (4.06 to 9.13)	6.93 (4.44 to 10.14)	0.19
2^nd^ assessment	5.93 (2.91 to 8.85)	5.76 (3.17 to 8.64)	0.51
Δ	−1.57 (−4.83 to 2.70)	−1.24 (−3.50 to 2.32)	
p^c^	0.06	0.09	
Ultra-processed (%E)			
1^st^ assessment	25.47 (19.27 to 32.79)	24.17 (19.08 to 32.29)	0.71
2^nd^ assessment	25.69 (18.65 to 34.87)	26.75 (18.87 to 35.27)	0.69
Δ	2.00 (−7.84 to 11.25)	3.74 (−5.86 to 12.86)	
p^c^	0.37	0.009	

%E: percentage of energy.

^a^ Data available for 294 women in the first assessment (159 women from the control group and 135 women from the intervention group) and 159 women in the second assessment (83 women from the control group and 76 women from the intervention group).

^b^ The p-values for the differences between the intervention and control groups were determined using the Mann-Whitney *U* test.

^c^ The p-values for the intragroup differences between the first and second assessments were determined using the Wilcoxon test.

At baseline, about 70% of the sample reported a sedentary lifestyle. An increase in CPA (min/week) occurred in the IG between assessments (Δ = 9.00; −30.00 to 70.00; p = 0.02). Additionally, a 6.9% increase occurred in the proportion of women in the IG meeting the weekly PA recommendation in the second assessment, whereas this increase only totaled 1.2% in the CG. Groups showed no difference (Table 3).

**Table 3:** Physical activity reported by the pregnant women, Ribeirão Preto, SP, Brazil, n = 301.

	Intervention^a^	Control^a^	p^b^
	Median (P25; P75)	Median (P25; P75)	
CPA (min/week)			
1^st^ assessment	56.00 (0.00 to 140.00)	60.00 (0.00 to 140.00)	0.48
2^nd^ assessment^e^	60.00 (0.00 to 180.00)	40.00 (0.00 to 120.00)	0.17
Δ	9.00 (−30.00 to 70.00)	0.00 (−73.75 to 30.00)	
p^c^	0.02	0.08	
LPA (min/week)			
1^st^ assessment	0.00 (0.00 to 0.00)	0.00 (0.00 to 0.00)	0.83
2^nd^ assessment	0.00 (0.00 to 0.00)	0.00 (0.00 to 0.00)	0.14
Δ	0.00 (0.00 to 0.00)	0.00 (0.00 to 0.00)	
p^c^	0.42	0.50	
CPA+LPA (min/week)			
1^st^ assessment	60.00 (0.00 to 180.00)	70.00 (0.00 to 183.75)	0.48
2^nd^ assessment	80.00 (30.00 to 202.25)	60.00 (0.00 to 170.00)	0.17
Δ	5.00 (−30.00 to 70.00)	00.00 (−70.00 to 40.00)	
p^c^	0.08	0.40	
Screen time (min/day)			
1^st^ assessment^f^	240.00 (120.00 to 412.50)	255.00 (180.00 to 420.00)	0.25
2^nd^ assessment^g^	300.00 (180.00 to 480.00)	300.00 (165.00 to 420.00)	0.90
Δ	9.00 (−97.50 to 157.50)	−22.50 (−120.00 to 120.00)	
p^c^	0.16	0.82	
			
	n (%)		p^d^
≥ 150 min (CPA +LPA)/week			
1^st^ assessment	40 (29.6)	48 (28.9)	0.89
2^nd^ assessment	35 (36.5)	31 (30.1)	0.34

CPA: commuting physical activity; LPA: leisure physical activity.

Note: LPA: In total, 32 pregnant women engaged in LPA at the first assessment and 36 at the second assessment. In the control group, only 17 women engaged in LPA at the first assessment and 14 at the second assessment. Regarding the intervention group, 15 women engaged in LPA at the first assessment, and 22 at the second assessment.

^a^ Data available for 301 women in the first assessment (comprising 166 women in the control group and 135 in the intervention group) and 197 women in the second assessment (including 101 women in the control group and 96 women in the intervention group).

^b^ The p-values for the differences between the intervention and control groups were determined using the Mann-Whitney U test.

^c^ The p-values for intragroup differences between the first and second assessments were determined using the Wilcoxon test.

^d^ The p-values for the association between practicing ≥ 150 minutes (CPA + LPA)/ week and the treatment groups were determined using the chi-squared test.

^e^ Data available for 96 women in the intervention group and for 100 in the control group.

^f^ Data available for 132 women in the intervention group and for 164 in the control group.

^g^ Data available for 97 women in the intervention group and for 101 in the control group.

## DISCUSSION

This unique study evaluated the effect of low-cost dietary counseling and PA promotion PHC can feasibly implement based on the NOVA food classification with overweight pregnant women. It found no effect of its intervention on the outcomes it explored between groups (thus, only within-group).

Previous interventions during pregnancy have effectively promoted healthy eating and LPA^14,32,33^, whereas others have failed to do so^34^ as various factors may influence adherence to new habits.

Although this study failed to confirm its hypothesis, it found an effect of decreasing %E from unprocessed and minimally processed foods and increasing %E from UPF between the evaluations in the CG. Recent studies have indicated that neighborhoods with high segregation, such as those in which the health units in this study are located, tend to have lower availability of minimally processed and unprocessed food shops and higher availability of UPF than more privileged areas^35,36^. Additionally, economically disadvantaged and segregated areas may have informal markets without adequate structures for selling minimally processed foods^37^. Furthermore, reduced family purchasing power and increased food prices in recent years have decrease the consumption of minimally processed and unprocessed foods^38^.

Thus, the intervention effectively curbed the increase in the consumption of ultra-processed foods and a decrease the consumption of in natura and minimally processed foods. However, its effect was too small to be detected in a direct comparison with the CG, possibly due to data variability and the small sample.

The intervention in this study differs from other successful ones due to its feasibility for implementation in PHC. Other interventions included an initial planning session and nutritional follow-up consultations providing a meal plan, food diary, recipe book, menu suggestions^32^, or even lifestyle interventions^33^.

Furthermore, two studies such as this one, conducted in PHC settings, found positive results such as a significant reduction in soft drink consumption, an increase in fish and vegetable consumption^14^, and a 50% reduction in the proportion of pregnant women with high weekly consumption of soft drinks and industrialized cookies^13^.

CPA increased in the IG between assessments. This study also found a 6.9% increase in the proportion of women meeting the weekly PA recommendation in its second assessment, whereas this increase only totaled 1.2% in the CG. High-intensity exercise interventions during pregnancy significantly reduced GWG^39^, although only in pregnant women with adequate pre-gestational BMI^40^.

Research indicates a high number of pregnant women with insufficient levels of PA (especially in their third trimester) due to the physiological changes of this condition. This trend worsened in the COVID-19 pandemic and its restrictions and isolation measures^41^. Other intervention studies also failed to prevent the spontaneous decline in PA in pregnant women^33,42^. This highlights a challenge for healthcare services and underscores the importance of integrated PA promotion programs in the PHC.

This study has some limitations. Its sample only consisted of overweight pregnant women, thus making it impossible to generalize its results to other BMI categories. Additionally, the COVID-19 pandemic caused follow-up losses, requiring a sample recalculation during this study. The original study protocol also required modifications to enable online interventions.

Furthermore, this study assessed LPA and CPA practices subjectively and ignored occupational and domestic PA. Accordingly, objective measurements of PA remain a challenge for PHC. However, self-reported measures are cost-effective, well-accepted, and can be applied across all practice domains.

PHC may have contaminated the sample in this study as its physical space is often limited for confidential interventions.

The strengths of this study specially include its novelty as this research found no other controlled randomized trials with low-cost interventions in PHC based on the NOVA food classification of the Brazilian Dietary Guidelines^23^. This makes this study unique and valuable for understanding the effectiveness of low-cost counseling programs that PHC settings can feasibly implement based on current recommendations for a healthy lifestyle during pregnancy.

## CONCLUSION

The intervention failed to affect the evaluated outcomes. However, the intragroup changes evinced that the CG consumed less minimally and unprocessed foods more UPF and that the IG increased their commuting-related PA.

This study was carried out during the COVID-19 pandemic, which may have influenced its findings. Therefore, future studies should re-test the suggested hypotheses.
